# Left ventricular hypertrophy in a contemporary cohort of autosomal dominant polycystic kidney disease patients

**DOI:** 10.1186/s12882-019-1555-z

**Published:** 2019-10-25

**Authors:** Huanwen Chen, Terry Watnick, Susie N. Hong, Barry Daly, Yongfang Li, Stephen L. Seliger

**Affiliations:** 1 Division of Nephrology, University of Maryland School of Medicine, 22 S. Greene street, N3W143, Baltimore, MD 21201 USA; 2 Division of Cardiology, University of Maryland School of Medicine, Baltimore, MD USA; 3 Department of Diagnostic Radiology, University of Maryland School of Medicine, Baltimore, MD USA

## Abstract

**Background:**

Patients with Autosomal Dominant Polycystic Kidney Disease (ADPKD) often develop hypertension in childhood or early adulthood. Although this could result in left ventricular hypertrophy (LVH), a major risk factor for cardiovascular morbidity and mortality, prior studies of LVH in ADPKD have yielded conflicting results. We estimated the prevalence of LVH using consensus echocardiography criteria and examined the independent association of ADPKD severity with LV mass in a contemporary cohort of ADPKD patients.

**Methods:**

Adults with ADPKD and eGFR> 15 ml/min/1.73m^2^ were enrolled in a single-center study. Left Ventricular Mass (LVM) was quantified using 2D echocardiography, and LVH was defined using gender-specific cut-points of LVM and LVM indexed to body surface area (LVMI) from consensus guidelines. Total Kidney Volume (TKV) was quantified using Magnetic Resonance Imaging, and GFR was estimated from serum creatinine using the CKD-Epi equation. Multiple linear regression was used to estimate the association of TKV and eGFR with LVM and LVMI, adjusting for potential confounders.

**Results:**

Among 126 participants (78% with hypertension), median age was 46 years, median eGFR 63 ml/min/1.73 m2, and median [IQR] systolic blood pressure was 125 [116–133] mmHg. Prevalence of LVH was 21.4% as defined by LVMI and was not significantly different (*p* = 0.8) between those with and without HTN, and was similar (21.4%) after excluding those (*N* = 21) with known cardiac disease. Greater TKV and lower eGFR were directly correlated with greater LVMI (*p* = .016 and *p* < .001, respectively). In multiple linear regression models accounting for potential confounders including blood pressure, greater TKV was positively associated with LVM ($\hat{\beta}$ =0.19, *p* = 0.04).

**Conclusions:**

In a contemporary cohort of ADPKD patients with well-controlled blood pressure, the prevalence of LVH is high, and ADPKD severity as reflected by TKV is independently associated with greater LV mass. These results may suggest a relationship between ADPKD pathophysiology and increased LV mass.

## Background

Hypertension is a highly prevalent and often early manifestation of autosomal dominant polycystic kidney disease (ADPKD), and a major risk factor for cardiovascular morbidity and mortality [1, 2]. There is evidence that clinical control of hypertension in ADPKD has improved over the last several decades [3]; however, whether this has resulted in a lower burden of cardiovascular disease is uncertain.

Increased left ventricular mass (LVM) and the development of left ventricular hypertrophy (LVH) are consequences of long-standing hypertension and strong predictors for risk of cardiovascular morbidity and mortality in the general population [4]. Prior studies have sought to investigate the relationship between ADPKD and LVH using different imaging modalities for LVM and different normative standards to define abnormal LVM and LVH [5, 6]. However, conflicting and inconclusive results have led to a lack of consensus on whether ADPKD patients are at increased risk of LVH, and whether the risk of LVH has decreased along with improved BP management.

In this observational study, we examined the prevalence of LVH among a cohort of adults with ADPKD using echocardiography, defining LVH with contemporary guidelines and normative data. We also investigated the relationship of ADPKD severity with greater LVM, accounting for other risk factors that determine LVM. We hypothesized that LVH remains highly prevalent in ADPKD and correlates with greater ADPKD severity as quantified by kidney volume and renal filtration function.

## Methods

### Study participants

Adults 18 years and older with ADPKD were enrolled into an observational cohort study at the Baltimore Polycystic Kidney Disease Center at the University of Maryland School of Medicine from June 2013 to March 2017. Patients with ADPKD as defined by Pei-Ravine criteria were included [7]. Individuals with prior kidney transplant, nephrectomy, and eGFR< 15 ml/min/1.73m^2^ were excluded. Patients were not excluded based on prior or current cardiovascular disease. The study protocol was approved by the University of Maryland, Baltimore Institutional Review Board.

### Study procedures

After written informed consent, personal and family medical histories were obtained by experienced nephrologists. Information on ADPKD complications, cardiac diseases (coronary heart disease, valvular heart disease, heart failure, or arrhythmia), and hypertension history and treatment were recorded. Patients with a past diagnosis of hypertension and patients who are currently on anti-hypertensive medications were considered to have a history of hypertension. Anthropometric measurements and demographics were collected. Blood pressure measurements were obtained 3 times in seated position after ≥5 min of rest; the average of these 3 measurements was utilized for analysis.

Blood and urine were collected after overnight fast. Serum creatinine was measured with an IDMS-traceable assay, and Glomerular Filtration Rate (GFR) was estimated using the creatinine-based CKD-Epi equation [8]. Albuminuria was expressed as the ratio of urinary albumin to creatinine in spot morning urine collection. Renal MRI was performed on willing participants without contraindications using a 3.0 Tesla Tim Trio system scanner (Siemens Medical) utilizing coronal volumetric T1-weighted, axial and coronal HASTE, and Coronal 3D TrueFISP gradient flow sensitive sequences. Images were exported to a 3D Workstation, and 3D volumes for each kidney were estimated and summed to estimate the total kidney volume (TKV). All TKV estimations were performed by the same experienced radiologist (B.D.) blinded to echocardiographic measurements and other patient information. Based on TKV, height, and age, all participants were classified by risk of PKD progression as defined by Irazabal et al. [9]

### Echocardiography

Echocardiograms were performed by an experienced sonographer using Philips iE33 xMATRIX ultrasound system (Philips Imaging, USA) and transferred to Synapse digital media (Fujifilm, USA). Because of limitations in the calculation of LVM using linear methods (M-mode imaging from 2D images), LVM was calculated with the two-dimensional method based on the area-length formula [10]. Image quality was evaluated in the 2D parasternal views, apical views and rated according to the identification of endocardium, and between the epicardium and pericardium. Since LVM is known to be associated with body size, LVM indexed (LVMI) to body surface area has been proposed to be a more appropriate metric for evaluating cardiovascular health. We used the method of Dubois [11] for estimation of BSA and indexing LVM. Both LVM and LVMI were used for analysis in this study. We elected to use BSA to index LVM in the primary analysis in accordance with ASE guidelines and consistent with prior cardiac imaging studies in ADPKD. However, as indexing to BSA can overestimate LVH in obese patients, in sensitivity analyses we indexed LV mass to an allometric function of height (height^2.7^), as recommended previously by de Simone et al. [12]

We defined LVH utilizing normative data in accordance with consensus guidelines of the American Society of Echocardiography [10]. As LVM and LVMI vary markedly between men and women, we utilized gender-specific thresholds to define abnormal LVM. ASE guidelines recommend an upper normal range of 200 g for men and 150 g for women for LVM, 102 g/m^2^ for men and 88 g/m^2^ for women for LVM index [10]. LVH subtype was defined by relative wall thickness (concentric> 0.42; eccentric ≤0.42) as per ASE guidelines. In a sensitivity analysis, we alternatively defined LVH using cut-points of LVM indexed to allometric height as recommended by de Simone et al. [12]

### Statistical analyses

Participant characteristics were presented as frequencies (for categorical variables) and medians and interquartile range (for continuous variables). Chi-squared analysis was used to compare LVH prevalence in the ADPKD cohort with normal populations, assuming 5% expected incidence of LVH in the normal population (based on an upper 95th percentile defining the upper limit of normal for LVM and LVMI). Associations of ADPKD severity, defined by total kidney volume and eGFR, with increased LVM and LVMI were evaluated using multiple linear regression. We adjusted for prior selected factors that would plausibly influence LVM including age, gender, race, body size, hypertension, clinically overt cardiac disease, and blood pressure. Using both indexed TKV and LVM in the same model may result in false associations driven by body size; therefore, in primary models we utilized unadjusted LVM as the primary (dependent) measure and unindexed TKV as a primary predictor variable, while including height and weight as adjustment covariates.

All variables were examined for normality, and bivariate and multivariate associations were performed using simple and multiple linear regression models respectively. As TKV, eGFR, LVM, and LVMI are represented in different units of measurement, we displayed associations in terms of both unscaled (β) and standardized ($\hat{\beta}$, per 1-SD unit difference) regression coefficients. Normality and homoscedasticity of residuals were evaluated and confirmed for all regression models using descriptive statistics. All statistical tests were performed on SPSS 20.0.

## Results

### Study population

One hundred twenty-six ADPKD patients were enrolled in our study. Of these, 114 patients received renal MRI, 7 patients declined, 3 patients had contraindications, and 2 patients were unable to perform the test due to large body habitus. Patients who did not receive an MRI were older and had significantly larger body size (*p* < 0.05), but did not have significantly different eGFR or LVM. Table 1 presents the characteristics of the study participants. 107 (85%) of participants were Caucasian, a diagnosis of hypertension was present in 98 (77.8%) of patients, and 21 (16.7%) had a diagnosis of prior cardiac disease. Among all participants, 80 (63.5%) were being treated with an ACE inhibitor or angiotensin receptor blocker at the time of echocardiography. Seventy-six percent of participants were Irazabal class1C-1E, representing high risk for renal progression.

**Table 1:** Patient characteristics

	N with complete data	N (Percentage) or Median (IQR)
Female Gender	126	76 (60.3%)
African American Race	126	14 (11.1%)
Age (years)	126	47 (35, 54)
Height (cm)	126	172.1 (164.5, 181.3)
Weight (kg)	126	80.4 (71.5, 91.7)
BMI (kg/m^2^)	126	26.8 (24.4, 30.3)
TKV (cc)	114	1792 (984, 2713)
Hemoglobin (g/dL)	124	13 (12, 14)
Urine Alb/Cr ratio (mg/g)	123	19.8 (11.3, 36.4)
eGFR (ml/min/1.73m^2^)	126	63 (43, 90)
Systolic BP (mmHg)	126	125 (116, 133)
Diastolic BP (mmHg)	126	78 (71, 84)
Heart Rate (/min)	126	62 (54, 68)
Irazabal ADPKD Class		
1A & 1B	114	24 (21.1%)
1C	114	38 (33.3%)
1D	114	30 (26.3%)
1E	114	22 (19.3%)
LVM (g)		
Male	50	179 (144, 205)
Female	76	140 (113, 172)
Both	126	159 (123, 187)
LVMI (g/m^2^)		
Male	50	85 (71, 97)
Female	76	79 (64, 87)
Both	126	81 (67, 92)
HTN Medications		
ACEI/ARB	126	80 (63.5%)
Diuretic	126	13 (10.3%)
Other Anti HTN Drug	126	31 (24.6%)
Medical History		
Hx of HTN	126	98 (77.8%)
Age of HTN Onset	126	35 (27, 46)
Duration of HTN	126	8 (1, 6)
Medical Hx of CVD	126	21 (16.7%)
Family Hx of CVD	126	61 (48.4%)

### Prevalence of LVH among ADPKD patients and comparison with normal populations

As shown in Table 2, the prevalence of LVH in the study sample was 40.5% when using LVM to define LVH, with 43.1% (*N* = 22) having concentric pattern and 56.9% (*N* = 29) having eccentric patter, based on relative wall thickness categories. When using LVMI to define LVH, prevalence was 21.4%, with 37% (*N* = 10) concentric and 63% [*N* = 17] eccentric. Among those with HTN, LVH defined by abnormal LVMI was present in 22 (22.4%) individuals, as compared to 5 (17.9%) among the minority of study participants without hypertension (*p* = 0.8). In a sensitivity analysis using allometric height to index LV mass, prevalence of LVH was 14.3% (*N* = 18).

**Table 2:** Prevalence of LVH in ADPKD population

	Based on LVM cut-off values			Based on LVMI cut-off values		
	*Men*	*Women*	*Both*	*Men*	*Women*	*Both*
All Patients	34.0% (17)	44.7% (34)	40.5% (51)	18.0% (9)	26.7% (18)	21.4% (27)
Irazabal 1A/B	66.7% (4)	27.8% (5)	37.5% (9)	50.0% (3)	5.6% (1)	16.7% (4)
Irazabal 1C	0.0% (0)	44.4% (12)	31.6% (12)	0.0% (0)	22.2% (6)	16.2% (6)
Irazabal 1D	64.3% (9)	43.8% (7)	53.3% (16)	35.7% (5)	25.0% (4)	30.0% (9)
Irazabal 1E	23.1% (3)	55.6% (5)	36.4% (8)	0.0% (0)	33.3% (3)	13.6% (3)

Data presented as Percentage (n)

After excluding the 21 participants (16.7% of cohort) with known overt cardiac disease, estimates of LVH prevalence (42.9% using LVM, and 21.9% using LVMI) remained very similar to the results from all participants (*p* = 0.8 and 1.0, respectively).

### Associations of ADPKD severity with LVM and LVMI

**Fig. 1:**
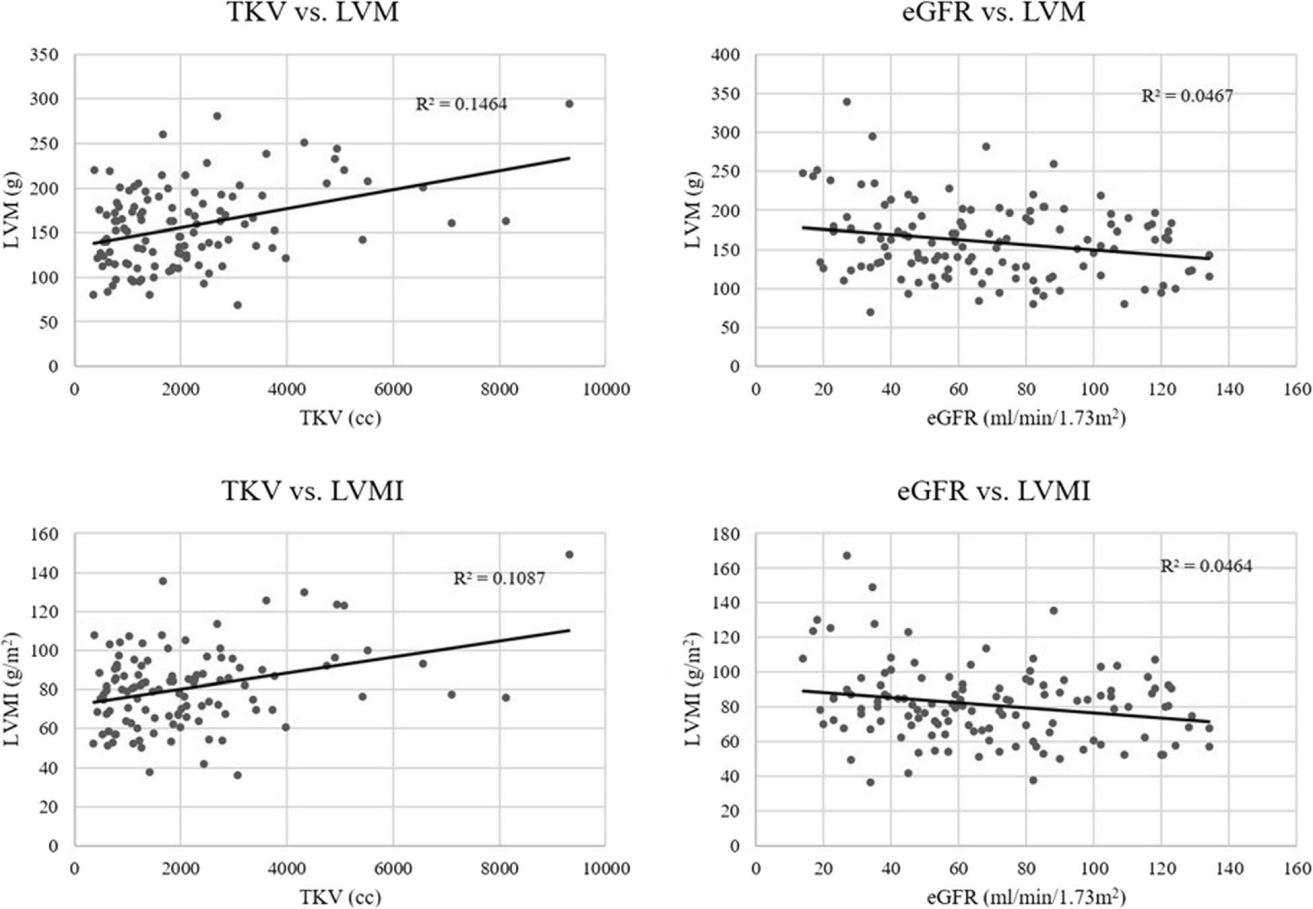
Unadjusted Associations of TKV and eGFR with LVM and LVMI

In unadjusted analyses, greater TKV and lower eGFR were both significantly associated with greater LVM and LVMI (Fig. 1, *p* < .05 for all associations). In multivariate analyses accounting for demographic and anthropometric characteristics (gender, race, age, height and weight), greater TKV was significantly associated with higher LVM ($\hat{\beta}$ =0.21, *p* = 0.02; Table 3). Neither age nor African-American race was associated with differences in LVM in our study population. Since elevated blood xpressure and prior cardiac disease are associated with LVM, systolic blood pressure, cardiac disease history, and a prior diagnosis of hypertension were included as additional adjustment covariates for further analyses. In these models, the association of greater TKV with LVM was only modestly attenuated and remained statistically significant ($\hat{\beta}$ =0.19, *p* = .04).

**Table 3:** Association of TKV with LVM

	Demographic Adjusted Model				Final Model		
	B	$\hat{\beta}$	Sig		B	$\hat{\beta}$	Sig
Female Gender	−14.10	−0.15	0.18	*Female Gender*	−12.60	− 0.14	0.23
AA Race	−11.44	− 0.08	0.33	*AA Race*	−14.49	−0.10	0.22
Age	0.33	0.10	0.26	*Age*	0.18	0.05	0.58
Height	0.34	0.07	0.53	*Height*	0.39	0.09	0.47
Weight	1.01*	0.35	< 0.001*	*Weight*	0.92*	0.32	0.001*
TKV (cm^3^)	0.006*	0.21	0.02*	TKV (cm^3^)	0.005*	0.19	0.04*
				*Systolic BP (mmHg)*	0.48	0.14	0.08
				*HTN diagnosis*	7.07	0.07	0.48
				*Cardiac Disease*	−4.73	−0.04	0.63
(Constant)	−0.64		0.99	*(Constant)*	−58.35		0.56
R^2^	0.35			*R* ^*2*^	0.38		

Significance: *p < 0.05

As shown in Table 4, eGFR was not statistically significantly associated with LVM either after adjustment for demographic factors and body size ($\hat{\beta}$ = − 0.10, *p* = 0.3) or after additional adjustment for SBP, hypertension and cardiac disease ($\hat{\beta}$ = − 0.06, *p* = 0.6). In this model, higher SBP was significantly associated with greater LVM ($\hat{\beta}$ =0.19, *p* = 0.02). We also regressed both TKV and eGFR on LVM (Table 5), and results showed that after accounting for demographic factors and body size, TKV was still significantly correlated with LVM ($\hat{\beta}$ =0.23, p = 0.02), while eGFR was not ($\hat{\beta}$ =0.05, *p* = 0.7). Similar results were found when additionally adjusting for cardiac disease, SBP and hypertension ($\hat{\beta}$ =0.23, p = 0.02 for TKV, and $\hat{\beta}$ =0.05, p = 0.7 for eGFR).

**Table 4:** Association of eGFR with LVM

	Demographic Adjusted Model				Final Model		
	B	$\hat{\beta}$	Sig		B	$\hat{\beta}$	Sig
Female Gender	−21.35	−0.22	0.05	*Female Gender*	−20.45	−0.21	0.06
AA Race	−5.91	−0.04	0.63	*AA Race*	−9.51	−0.06	0.43
Age (years)	0.44	0.12	0.23	*Age (years)*	0.35	0.10	0.34
Height (cm)	−0.017	−0.004	0.97	*Height (cm)*	0.05	0.01	0.93
Weight (kg)	0.98*	0.34	< 0.001*	*Weight (kg)*	0.88*	0.31	0.001*
eGFR (mL/min/1.73m^2^)	−0.15	−0.10	0.33	*eGFR (ml/min/1.73m* ^*2*^ *)*	−0.08	−0.06	0.60
				*Systolic BP (mm Hg)*	0.67*	0.19	0.02*
				*HTN diagnosis*	6.49	0.06	0.54
				*Cardiac Disease*	2.99	0.02	0.77
(Constant)	84.61		0.41	*(Constant)*	−7.88		0.94
R^2^	0.27			*R* ^*2*^	0.31		

Significance: *p < 0.05

**Table 5:** Association of TKV and eGFR with LVM

	Demographic Adjusted Model				Final Model		
	B	$\hat{\beta}$	Sig		B	$\hat{\beta}$	Sig
Female Gender	−13.66	−0.15	0.20	Female Gender	−11.69	−0.13	0.27
AA Race	−11.89	− 0.08	0.31	*AA Race*	−15.45	−0.11	0.19
Age	0.42	0.12	0.24	*Age*	0.30	0.09	0.42
Height	0.37	0.08	0.50	*Height*	0.45	0.10	0.41
Weight	1.01*	0.34	< 0.001*	*Weight*	0.88*	0.30	0.003*
TKV (cm^3^)	0.006*	0.23	0.02*	TKV (cm^3^)	0.006*	0.21	0.03*
eGFR (ml/min/1.73m^2^)	0.07	0.05	0.67	*eGFR (ml/min/1.73m* ^*2*^ *)*	0.11	0.08	0.50
				*Systolic BP (mmHg)*	0.47	0.14	0.09
				*HTN diagnosis*	9.33	0.09	0.38
				*Cardiac Disease*	−4.88	−0.04	0.62
(Constant)	−15.73		0.88	*(Constant)*	−82.03		0.44
R^2^	0.35			*R* ^*2*^	0.38		

Significance: *p < 0.05

Since eGFR is derived from an equation as a function of age, race, and gender (factors which may relate independently to LVM), we further evaluated the association of serum creatinine (rather than eGFR) with LVM. Results showed that while serum creatinine was significantly correlated with LVM when accounting for demographic factors and body size ($\hat{\beta}$ =0.18, *p* = 0.04), this relationship was attenuated after additional adjustments for SBP, cardiac disease and hypertension ($\hat{\beta}$ =0.14, *p* = 0.13).

We also performed sensitivity analyses regressing on LVMI (indexed to BSA) rather than unindexed LVM, without further adjustment for body size as covariates. As shown in Tables 6 and 7, results were similar as with unindexed LVM, with significant associations of greater TKV with greater LVMI after adjustment for demographic factors, HTN diagnosis, SBP, and cardiac disease ($\hat{\beta}=0.22$, *p* = .04). This association remained significant when additionally considering eGFR ($\hat{\beta}=0.25$, *p* = .03, Table 8), while eGFR was not associated with greater LVMI with or without consideration of TKV (Table 7). In sensitivity analyses, associations of TKV with LVMI where similar ($\hat{\beta}=0.18\Big)$ when allometric height was used to index LV Mass rather than BSA. Serum creatinine was also not significantly associated with LVMI after adjusting for demographic factors, HTN diagnosis, cardiac disease, and SBP.

**Table 6:** Association of TKV with LVM

	Demographic Adjusted Model				Final Model		
	B	$\hat{\beta}$	Sig		B	$\hat{\beta}$	Sig
Female Gender	−5.96	−0.15	0.13	*Female Gender*	−4.80	−0.12	0.23
AA Race	−5.31	− 0.08	0.37	*AA Race*	−7.46	−0.11	0.21
Age (years)	0.16	0.10	0.29	*Age (years)*	0.07	0.05	0.65
TKV (cm^3^)	0.003*	0.25	0.01*	*TKV (cm* ^*3*^ *)*	0.003*	0.22	0.04*
				*Systolic BP (mmHg)*	0.27	0.18	0.05
				*HTN diagnosis*	4.51	0.09	0.35
				*Cardiac Disease*	−2.85	−0.05	0.57
(Constant)	71.04*		< 0.001*	*(Constant)*	38.19*		0.03*
R^2^	0.14			*R* ^*2*^	0.18		

Significance: *p < 0.05

**Table 7:** Association of eGFR with LVM

	Demographic Adjusted Model				Final Model		
	B	$\hat{\beta}$	Sig		B	$\hat{\beta}$	Sig
Female Gender	−7.41	−0.17	0.06	*Female Gender*	−6.62	− 0.15	0.09
AA Race	−2.32	− 0.03	0.70	*AA Race*	−4.61	−0.07	0.45
Age (years)	0.28	0.17	0.12	*Age (years)*	0.23	0.14	0.21
eGFR (ml/min/1.73m^2^)	−0.06	−0.09	0.41	*eGFR (ml/min/1.73m* ^*2*^ *)*	−0.03	−0.04	0.74
				*Systolic BP (mm Hg)*	0.35*	0.23	0.01*
				*HTN diagnosis*	3.80	0.07	0.46
				*Cardiac Disease*	1.52	0.03	0.77
(Constant)	77.43		< 0.001*	*(Constant)*	29.60		0.17
R^2^	0.09			*R* ^*2*^	0.15		

Significance: *p < 0.05

**Table 8:** Association of TKV and eGFR with LVM

	Demographic Adjusted Model				Final Model		
	B	$\hat{\beta}$	Sig		B	$\hat{\beta}$	Sig
Female Gender	−5.90	−0.14	0.14	*Female Gender*	−4.57	−0.11	0.15
AA Race	−5.61	−0.09	0.34	*AA Race*	−8.07	−0.12	0.25
Age (years)	0.21	0.14	0.24	*Age (years)*	0.14	0.09	0.18
TKV (cm^3^)	0.003*	0.27	0.01*	*TKV (cm* ^*3*^ *)*	0.003*	0.25	0.03*
eGFR (ml/min/1.73m^2^)	0.04	0.06	0.61	*eGFR (ml/min/1.73m* ^*2*^ *)*	0.06	0.10	0.44
				*Systolic BP (mmHg)*	0.27	0.18	0.06
				*HTN diagnosis*	5.57	0.12	0.27
				*Cardiac Disease*	−2.92	−0.05	0.56
(Constant)	65.19*		< 0.001*	*(Constant)*	29.66		0.15
R^2^	0.14			*R* ^*2*^	0.19		

Significance: *p < 0.05

## Discussion

Among 126 adults with ADPKD, we found that left ventricular hypertrophy as assessed by 2D echocardiography was present among roughly 20% of patients, and this prevalence was not significantly different between subjects with and without hypertension. We further observed a linear association of greater total kidney volume with greater left ventricular mass, even after accounting for blood pressure, body size, and other risk factors. This observational study provides a contemporary analysis of the relationship between left ventricular mass and ADPKD. Our results confirm some previous findings and provide several key new insights.

### Prevalence of LVH

Comparison of LVH prevalence across different studies is challenging because of differences in imaging techniques and LVH definitions, as well as variability in the characteristics of study participants. An early study from Chapman et al. reported a 41% prevalence of LVH using transthoracic echocardiography, with a prevalence of 48% among those subjects with hypertension [5]. The prevalence of LVH in our study was considerably lower, including among those with hypertension. This was despite using less stringent cutoffs to define LVH (102 g/m^2^ in men and 88 g/m^2^ in women in the present study, versus 125 g/m^2^ in men and 110 g/m^2^ in women in Chapman et al).

Improved blood pressure control may account for at least a part of the difference between our data and those presented in Chapman et al. Mean systolic and diastolic blood pressure in our participants were about 10 mmHg and 12 mmHg lower, respectively, compared with the cohort studied by Chapman et al. Alternatively, different definitions of LVH could explain the lower prevalence. We utilized a consensus definition which defined the upper limit of LVM based on gender-stratified normative values; in contrast, the prior study utilized a local healthy control population, among whom 16% was reported to have LVH.

In contrast, a more recent analysis of data from the HALT clinical trial, utilizing cardiac MRI [6], reported a markedly lower prevalence of LVH at 3.9% based on unindexed LVM and 0.9% based on LVMI. In that study, LVH was defined as >95th percentile expected based on gender and height among healthy adults in the multicenter MESA cohort study. Of note, participants in the HALT analysis were younger, had hypertension of recent onset, and all had eGFR> 60 ml/min/1.73m^2^; however, mean blood pressure in the HALT cohort was similar to that observed in the present study. Whether patient factors, differences in imaging modality, or the selection of the normative reference data explain the marked differences in LVH prevalence compared to our study and that of Chapman et al. is uncertain.

The prevalence of LVH in our ADPKD population is substantial when using gender-stratified LVM thresholds recommended by the ASE; however, there is debate regarding the appropriateness of indexing or stratifying LVH definitions based on age and race. Current clinical practice as consistent with ASE guidelines do not utilize age and race stratifications. In the present study, we found no independent associations of older age with greater LVM, supporting the use of age-independent LVH definitions for our primary analysis.

There is likewise ongoing debate about the most appropriate methods for indexing LV mass for body size. Although prior studies of LVH in ADPKD used BSA to index LVM - and guidelines from the ASE likewise recommend BSA indexing – this may lead to an over-estimation of LVH in obese individuals. As expected, the prevalence of LVH was lower (14.3% vs. 21.4%) when LVMI was defined by indexing to allometric height rather than BSA. However, caution should be used when interpreting this LVH prevalence, considering the less robust normative data available for LVMI indexed to allometric height.

We subclassified a majority of LVH cases (63%) as eccentric. We are not aware of prior reports on the relative LV geometry observed in LVH cases in ADPKD. We have previously reported a similar frequency (71%) of eccentric pattern of LVH among older community-dwelling adults with and without hypertension [13]. Prior studies of experimental models of LV remodeling have suggested that eccentric LVH develops in response to chronic volume overload and concentric LVH in response to chronic pressure overload [14]. Given the small number of total LVH cases, we were not able to separately examine the correlates of eccentric vs. concentric LVH in this study.

### Association of ADPKD severity with increased LVM or LVMI

We found significant associations of greater total kidney volume with greater left ventricular mass even after accounting for differences in age, gender, and body size. Furthermore, these associations were not explained by higher blood pressure, the presence of diagnosed/treated hypertension, nor known cardiac disease. Prior studies also reported significant unadjusted associations of kidney volume with greater LVM by echocardiography or Cardiac MRI, but these associations were not observed after accounting for other patient characteristics including renal function, blood pressure, age, and gender [6]. Total Kidney Volume is a widely utilized and reproducible measure of ADPKD severity, highly prognostic for progression to ESRD, [15, 16] and has been used as a surrogate disease marker in interventional clinical trials [17, 18]. In the present study, greater TKV was associated with higher LVM and LVMI even after accounting for eGFR.

In contrast, although lower eGFR was modestly associated with greater LVM in unadjusted analyses, no associations were observed after accounting for demographic factors and body size. Likewise, Perrone et al. [6] found no relationship between eGFR and LVMI by MRI in the HALT population with initial eGFR> 60 ml/min/1.73m^2^ in neither unadjusted nor adjusted analyses. However, the study by Chapman et al., [5] conducted before widespread use of estimating GFR equations, found marginally significant (*p* = .05) associations of higher serum creatinine with greater LVMI, although the magnitude of this association was not reported. As serum creatinine concentrations and even estimating equations for GFR are insensitive for detecting differences in true GFR when GFR > 60 ml/min/1.73m^2^, the relationship of eGFR and serum creatinine with LVM may only be apparent among ADPKD patients with more advanced renal functional impairment. Furthermore, detectable decline in eGFR may be a later manifestation of ADPKD, observable only after substantial nephromegaly has occurred.

Of note, Bansal et al. recently reported modestly greater LVMI associated with eGFR 60–75 ml/min/1.73m^2^, when using cystatin C-based eGFR equations, among 2400 younger adults without ADPKD. These results suggest a possible association of mild kidney disease with greater LVMI among those without ADPKD. The use of a less sensitive creatinine-based estimating equation and the smaller sample size in our study may have precluded detecting more modest associations [19].

Our findings of a notably greater prevalence of LVH in ADPKD patients, and significant independent associations of TKV with greater LV mass, support the hypothesis of a relationship between ADPKD and progressive hypertrophy of the left ventricle. Several mechanisms have been proposed to account for this relationship. The relationship between ADPKD and higher blood pressure has been extensively documented in both adults and children [20–23], and higher blood pressure have been shown to be associated with increased LVM in ADPKD patients [5, 6, 24, 25]. Our findings of linear associations of blood pressure with greater LVM are consistent with these past reports, and support the hypothesis that high blood pressure in ADPKD may be a major contributor to increased LVM in this population. However, our observation that greater TKV is associated with higher LVM and LVMI even after accounting for BP control suggests that additional, more disease-specific mechanisms may be present. Findings that support this hypothesis have also been reported in the past, showing that LVMI is higher in normotensive ADPKD patients compared to healthy controls despite similar BP measurements on 24-h monitoring [24]. ADPKD patients are at an increased risk of cardiac valvular dysfunction such as mitral valve dysfunction, [26] which may contribute to the development of LVH [27]. Furthermore, it has also been proposed that an altered myocardial phenotype may be present in ADPKD, making increased LVM a possible primary manifestation of ADPKD. For example, studies in murine models have demonstrated that reduced polycystin-2 expression may result in cardiac remodeling and dysfunction (though not specifically hypertrophy), in part mediated by altered intracellular calcium signaling [28]. In patient cohorts, a higher than expected frequency of primary cardiomyopathies including idiopathic dilated cardiomyopathy and obstructive cardiomyopathies has been reported [29]. Furthermore, intra-renal activation of the renin-angiotensin aldosterone system is an early complication of ADPKD [30]. Such activation may lead to adverse cardiac remodeling through both blood pressure-dependent and pressure-independent mechanisms. Pharmacological blockade of the RAAS can lead to regression or improvement in LVH in adult and pediatric ADPKD populations [31, 32], including a recent report by Dad et al. showing that intensive blood pressure control can lead to reduction of LVMI, especially in patients with higher baseline BP measurements [33]. Of note, in the present study, nearly 2/3^rds^ of participants were on current treatment with RAAS inhibitors.

It is also important to note that the association between TKV and LVM, though significant, is only modest to moderate in magnitude. Thus, while ADPKD severity alone can impact LVM independent of blood pressure and other factors, and as such suggest a biological relationship of ADPKD severity with LVM in adult ADPKD patients, the clinical implications of this association may be modest. Furthermore, while LVH is potentially reversible with proper BP control, LVH may still remain as a complication of past uncontrolled hypertension (not assessed in this study). Therefore, we do not suggest that TKV can be considered as a reliable predictor for the presence or absence of LVH, nor that the relationship between ADPKD and LVH is completely independent of blood pressure.

### Limitations

This was a single-center study, although conducted within a tertiary subspecialty PKD center with a large geographic referral base in the Mid-Atlantic US. Results may not be generalizable to other regions. The study cohort was largely Caucasian, with insufficient number of ethnic/racial minorities to examine LVH prevalence and correlations separately in these subgroups. This study had only a moderate sample size, which makes analyses vulnerable to imprecisions in LVH estimates and leads to limited power to detect smaller associations of patient factors with LVM. Echocardiography is the most widely used method in clinical practice for the detection and quantification of LVH, but this imaging modality has substantially greater measurement error than cardiac MRI. Blood pressure measurements were acquired in a single office setting as opposed to 24 h measurements and may not reflect overall blood pressure control. Past history and duration of uncontrolled hypertension, which may lead to increased LVM, were not assessed. We attempted to minimize measurement variability between subjects by utilizing a central echocardiography laboratory and standardized technique for all participants. To the extent that measurement error would be expected to occur at random with regards to PKD severity, such random error would be expected to lead to a bias towards the null hypothesis of no association between TKV and LVM/LVMI.

## Conclusions

In this observational cohort study, we found that adult ADPKD patients have a higher prevalence of LVH as evaluated by echocardiography compared to the normal healthy population, and that ADPKD disease severity as measured by TKV is significantly correlated with increased LVM after accounting for well-known covariates such as gender, race, age, body size, blood pressure, cardiac disease, and history of hypertension. Further investigations are needed to more clearly validate these associations and elucidate underlying mechanisms.

## Data Availability

The datasets used and/or analysed during the current study are available from the corresponding author on reasonable request.

## Abbreviations

ADPKD: Autosomal Dominant Polycystic Kidney Disease
BMI: Body Mass Index
HTN: Hypertension
LVH: Left Ventricle Hypertrophy
LVM: Left Ventricle Mass
LVMI: Left Ventricle Mass Index
MRI: Magnetic Resonance Imaging
TKV: Total Kidney Volume
